# Mocking enactments: a case study of multimodal stance-stacking

**DOI:** 10.3389/fpsyg.2024.1379593

**Published:** 2024-04-02

**Authors:** Clarissa de Vries, Fien Andries, Katharina Meissl

**Affiliations:** ^1^Department of Linguistics, Faculty of Arts, KU Leuven, Leuven, Belgium; ^2^Department of Linguistics, Faculty of Arts, KU Leuven, Antwerp, Belgium

**Keywords:** mocking, stance, enactment, depiction, multimodality, Dutch, German, Flemish Sign Language

## Abstract

Although research into multimodal stance-taking has gained momentum over the past years, the multimodal construction of so-called stacked stances has not yet received systematic attention in the literature. Mocking enactments are a prime example of such complex social actions as they are layered both interactionally and stance-related, and they rely significantly on the use of bodily visual resources, depicting rather than describing events and stances. Using Du Bois’ Stance Triangle as a framework, this study investigates mocking enactments as a case study to unravel the multimodal aspects of layered stance expressions. Drawing on three data sets—music instruction in Dutch, German, and English, spontaneous face-to-face interactions among friends in Dutch, and narrations on past events in Flemish Sign Language (VGT)—this study provides a qualitative exploration of mocking enactments across different communicative settings, languages, and modalities. The study achieves three main objectives: (1) illuminating how enactments are used for mocking, (2) identifying the layers of stance-taking at play, and (3) examining the multimodal construction of mocking enactments. Our analysis reveals various different uses of enactments for mocking. Aside from enacting the target of the mockery, participants can include other characters and viewpoints, highlighting the breadth of the phenomenon under scrutiny. Second, we uncover the layered construction of stance on all axes of the Stance Triangle (evaluation, positioning, and alignment). Third, we find that mocking enactments are embedded in highly evaluative contexts, indexed by the use of bodily visual resources. Interestingly, not all mocking enactments include a multimodally exaggerated depiction, but instead, some merely allude to an absurd hypothetical scenario. Our findings contribute to the growing body of literature on multimodal stance-taking, by showing how a nuanced interpretation of the Stance Triangle can offer a useful framework for analyzing layered stance acts.

## 1 Introduction

Mocking is a form of playful exchange, in which stance takes center stage. During mocking, participants express a stance on a serious layer that can be heightened, diminished, or inverted on a non-serious layer. In other words, they stack stances onto each other (Dancygier, 2012; Andries et al., 2022). One resource that is eminently suitable for the expression of stances on different interactional layers is enactment as it allows interactants “to ‘construct’ actions and dialog in order to ‘show’ characters, events, and points of view” as they combine “bodily movements, postures, and eye gaze” (Hodge and Ferrara, 2014, p. 373). In enactments, participants may represent a stance of the character they enact, i.e., showing what this character feels and thinks, what their opinion is, what they know, or present their own stance, from a narrator’s point of view. Moreover, multiple stances may be combined and stacked onto each other, as is the case in mocking enactments. Consider the following example from one of the data sets of the current study. Three friends (Emma, Jana, and Alyssa) are talking about a time when Jana went away for a weekend with a group of scouts and stayed in a house in the woods that had no keys (so that the house could not be locked). Jana enacts the landlady of the house, who did not consider this to be a problem. Accompanied by multiple shoulder shrugs, palm up open hand gestures, and head shakes, she says “oh gosh, well, throughout the years, all the keys just got lost.” Thereby, Jana mocks the indifferent attitude of the landlady toward this issue.

As illustrated by this example, both in enactments and during mocking, participants leverage an array of both lexical and non-lexical resources across various modalities. However, the research on multimodal aspects of how enactments are used in layered stance expressions remains limited so far. In this study, mocking enactments serve as a case study to unravel multimodal dynamics of stance-stacking and the underlying mechanisms at play. We zoom in on mocking enactments as a case of stance-taking as a multimodal, polysemiotic^1^ phenomenon, without a predetermined hierarchy of semiotic resources; instead, they are employed flexibly, contingent upon the interactional context. In this exploration, we use the Stance Triangle (Du Bois, 2007) as a conceptual framework and consider three dimensions of stance: evaluation of the stance object, positioning of stance subjects, and their alignment. Our focus is on dissecting and understanding the intricate dynamics of mocking enactments, shedding light on the nuanced layers of stance inherent in these communicative acts.

The current exploratory study pursues three primary objectives. First, we aim to illuminate the ways in which enactments are used for mocking. Second, we identify the layers of stance-taking operative across all components of the Stance Triangle. Third, we examine the multimodal construction of these enactments and layers of stance. To achieve these aims, the study draws on corpora from three settings: (1) music instruction in Dutch (Schrooten and Feyaerts, 2020), as well as German and English (MuTh, 2021), (2) spontaneous face-to-face interactions among friends in Dutch (Brône and Oben, 2015; de Vries et al., n.d.), (3) narrations of past events from the corpus Flemish Sign Language (VGT, Van Herreweghe et al., 2015). The combination of three data sets allows for a broad and yet qualitative approach to mocking enactments in different communicative settings as well as languages in different modalities and from different communities.

In Section 2, we delve deeper into the multimodal analysis of stance-taking, mocking, and its relation to stance-stacking, as well as enactment as a polyphonic communicative device. Section 3 contains our methodological approach, along with a presentation of the three different data sets used in the study. The analysis is presented in Section 4, starting with an emphasis on the evaluation process of the Stance Triangle. Subsequently, we shift our focus to the theme of positioning, and finally, we scrutinize alignment. The study’s findings are synthesized and discussed in Section 5. A conclusion, encapsulating the key insights and implications, is presented in Section 6.

## 2 Theoretical background and rationale

### 2.1 Multimodal stance-taking

One of the primary functions of language and interaction more broadly is to communicate how we feel about the world around us. Throughout our daily lives, the negotiation of our attitudes is ubiquitous: “do not eat that”; “did you like the show?”; and “I do not know how this works,” are just a few everyday examples of the linguistic negotiation of stance in different dimensions (in this case, deontic, affective, and epistemic, respectively).

**Figure 1:**
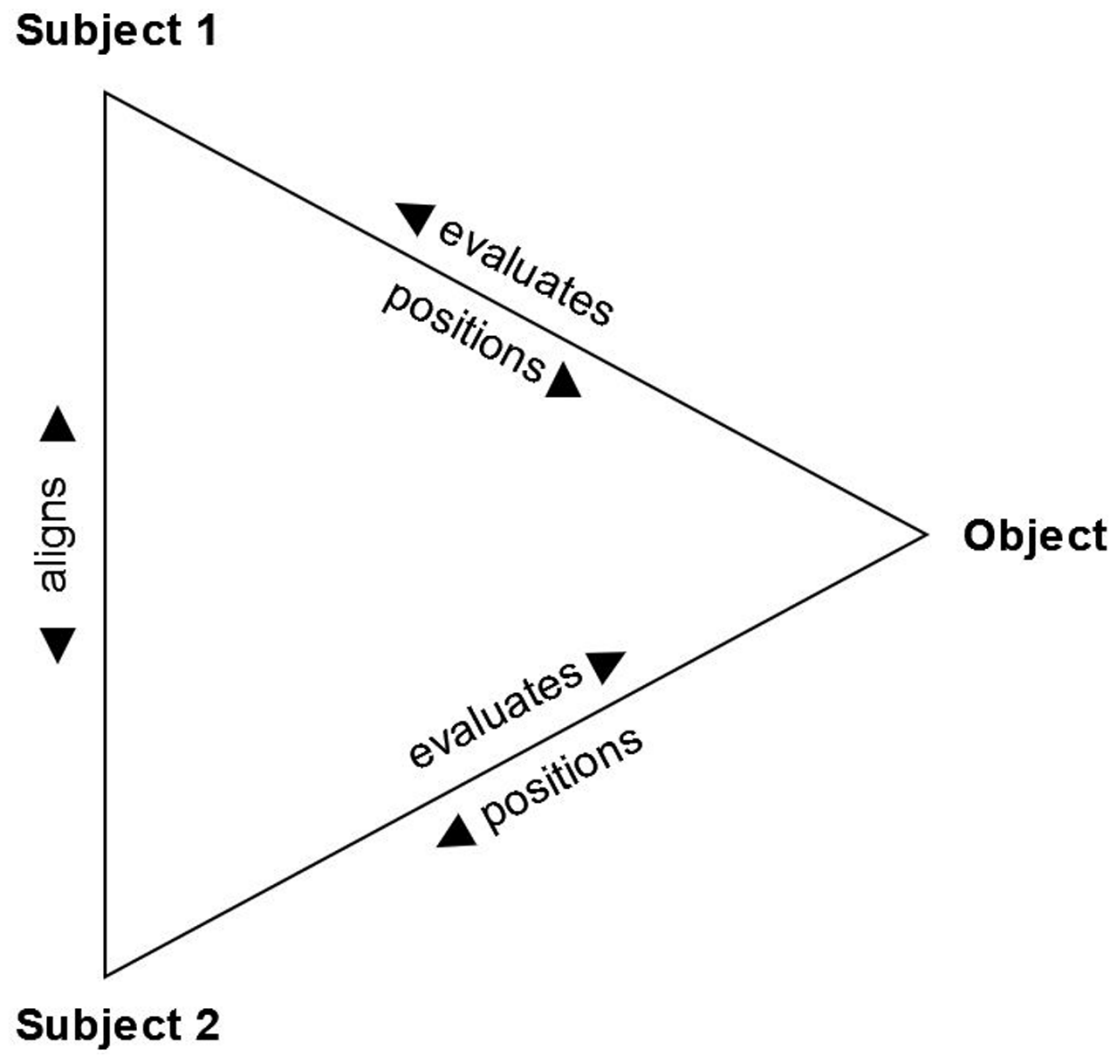
The stance triangle (adapted from Du Bois, 2007, p. 163).

In the current study, we draw on the influential analytical framework of the Stance Triangle, by Du Bois (2007), as it presents a broad view on stance-taking and brings together cognitive and interactional dimensions. Three questions at the core of the framework direct us to the components of stance-taking: “*(1) Who is the stancetaker? (2) What is the object of stance? (3) What stance is the stancetaker responding to?”* (Du Bois, 2007, p. 146). These questions connect stance subjects and stance objects through three processes: evaluation, positioning, and alignment. The processes and their relation to stance subjects and objects can be visualized in a triangle (see Figure 1).

As can be seen in Figure 1, the result is a dynamic and intersubjective process of stance negotiation, which Du Bois captures in the following definition: “Stance is a public act by a social actor, achieved dialogically through overt communicative means, of simultaneously evaluating objects, positioning subjects (self and others), and aligning with other subjects, with respect to any salient dimension of the sociocultural field” (Du Bois, 2007, p. 163). More recently, Iwasaki (2022) argued for an elaboration of the Stance Triangle so as to take into account the evolvement of stances across longer stretches of time. While the Stance Triangle can be considered as a ‘snapshot’ of the stance at a given moment in interaction, Iwasaki (2022, p. 15) highlights that “all aspects of the stance activity are in constant motion and emerge across several turns along the temporal trajectory during an interaction”.

Furthermore, although the Stance Triangle is modality agnostic in theory, traditionally, the focus of stance-taking research has been on lexico-grammatical and to a lesser extent prosodic means (Biber and Finegan, 1989; Couper-Kuhlen, 2012; Wang et al., 2022). Over the past years, interest in multimodal research on stance-taking has increased (Andries et al., 2023). Research in this domain highlighted that the whole body can be used to take a stance, with individual semiotic resources operating flexibly depending on the interactional needs. The compound shrug is a prototypical example, as it consists of a varying constellation including raised shoulders, palm up gestures, head tilts, and raised eyebrows, and can express multiple forms of distancing, such as obviousness, indifference, or incapacity (Debras, 2017), as we saw in the example above with the lost keys. Furthermore, the affordances of bodily visual resources are highly suitable to monitor and negotiate the temporal development of stance as they allow for ensuring fit responses (Pillet-Shore, 2020), negotiating alignment (Iwasaki, 2015), and synchronizing the expression of stance (Pfänder and Couper-Kuhlen, 2019), without disrupting the flow of conversation. Finally, and of relevance for the current study, multiple semiotic resources can be used simultaneously not only in function of one holistic “gestalt” (Mondada, 2014) such as the shrug but also in order to allow signers/speakers to present multiple stances or viewpoints at the same time (Kärkkäinen, 2012; Soulaimani, 2018; Vandenitte, 2022).

Although there is considerable evidence for the inherent multimodal nature of stance-taking (Andries et al., 2023), there are still many open questions. One aspect that has not received systematic attention yet concerns the construction of so-called stance-stacks (Dancygier, 2012) in which multiple stances are communicated simultaneously. The current study therefore aims to scrutinize this phenomenon, by looking at a case study of layered stance-taking that has been surprisingly understudied: the use of mocking enactments. In what follows, we will briefly review the literature on mockery (Section 2.2) and enactments and depictions (Section 2.3) before presenting the research questions and aims of the current study.

### 2.2 Mocking as stance-stacking

Mocking concerns a playfully constructed negative stance toward something of relevance to a target. As the term itself implies, mocking involves some kind of pretense or imitation. As such, it can be considered a *staged communicative act* (Clark, 1996). Staged communicative acts involve a layered construction of actions, in which language users engage in a *joint pretense* (Clark and Gerrig, 1984). A signer/speaker addresses an addressee A, while at the same time pretending to be another signer/speaker S′, addressing another addressee A′ (Clark and Gerrig, 1984, p. 122). The contrast between the demonstrated and actual situation gives rise to the mocking, which the addressee is expected to recognize and appreciate. For instance, in the example with the missing keys from the introduction, Jana enacts the landlady’s stance and thereby pretends to be indifferent about the lost keys. Simultaneously, Jana’s own stance—that this is unsafe—becomes apparent, resulting in a stance-stack with a mocking quality (Janzen, 2022).

Of particular relevance in the context of stance-taking is the fact that mocking is directed at a target, which can be the signer/speaker themselves, another participant in the interaction, or a non-co-present third party. Depending on the target of the mockery and the interactional setting, mocking can fulfill a wide range of functions, including bringing shared amusement (Yu, 2013), sanctioning transgressions (Drew, 1987), managing errors in instruction (Poggi, 2015), and building relationships (Boxer and Cortés-Conde, 1997). Arguably, the non-serious layer allows participants to deal with potentially sensitive issues. Or, in the words of Holt (2013, p. 89): “Introducing non-seriousness does not mean that the serious sequential implications are completely swept away. Rather, it enables an intertwining of strands as serious matters are dealt with more and less seriously, allowing for a more delicate and implicit touch”. For instance, during orchestra rehearsals, conductors can use mocking to point to errors in the musicians’ performance, while maintaining a friendly atmosphere (Poggi, 2015).

Crucially, mocking is constructed and negotiated in interaction, which can be quite complex given its layered nature. Bodily visual practices have often been described to play a large role in this regard (Clark, 1996, p. 370), and in what follows we will present an overview of research into the multimodal construction of mocking. As research on multimodal aspects of mockery itself is rather limited, we also draw on work on related forms of staged communicative acts, including irony and humor.

The intuitively most obvious bodily visual practices used to construct non-seriousness are laughter and smiling. Turn-final laughter, for instance, can function to frame an utterance as humorous or non-serious (Holt, 2013), as well as to invite other participants to laugh along and thereby affiliate with an interlocutor (Jefferson et al., 1987). The closely related resource of smiling has traditionally also been associated with non-seriousness. Recent studies confirm that an increase in smiling intensity (by both speakers and addressees) is associated with the use of humor (Gironzetti, 2022) and irony (González-Fuente et al., 2015). However, the relationship of smiling and laughter with affiliation and non-seriousness is not straightforward. Laughter occurs both in the context of ‘laughing with’ and ‘laughing at’ (Glenn, 2003), or both at the same time (Clift, 2016), and smiling, similarly, is also found to be associated with embarrassment or nervosity (Ambadar et al., 2009). Furthermore, the presence of laughter or smiling is not a prerequisite for the successful performance of humor (Priego-Valverde and Rauzy, 2023).

Other resources can be ascribed to the staging of an exaggerated scenario or otherwise highlighting the incongruence between reality and expectation. Earlier work on verbal cues in written discourse mentions the use of extreme case formulations or overstatements (Norrick, 2004) to convey humorous or ironic intent, as well as shifts in register or other formulaic markers (Burgers, 2010). The multimodal staging of mocking is often connected to its stance-related aspects (see Section 2.1 for a discussion of multimodal markers of stance). Such realizations include covering the face with the hands in response to a laughable, throwing the head back, or shaking the torso and shoulders (Ford and Fox, 2010). Gaze aversion has frequently been mentioned as a cue to convey ironic intent by speakers (Caucci and Kreuz, 2012; González-Fuente et al., 2015; Gironzetti, 2022) and can also be linked to a stance-taking role, namely, displaying disalignment (Haddington, 2006), or in the context of self-mockery, embarrassment (Yu, 2013).

Multimodal complements for exaggeration and overstatements can be found, for example, in the case of parodies, where a “distorted imitation” is performed, “in which the target’s traits are exaggerated and made grotesque” (D’Errico and Poggi, 2016, p. 4), a finding also reported in Ford and Fox’s (2010) analysis of constructing laughables. The whole gamut of semiotic resources can be crucial when it comes to staging or animating characters as part of a mocking event. In what follows, we dig deeper into the design and use of enactment.

### 2.3 Enactment and depiction

Before we turn to the specific role of enactment for stance-taking and mocking, let us introduce the phenomenon in general. Enactment refers to “signers and speakers combining bodily movements, postures, and eye gaze to ‘construct’ actions and dialog in order to ‘show’ characters, events, and points of view” (Hodge and Ferrara, 2014, p. 373).

As such, enactments fulfill the function of *depicting* meaning, as distinguished from the other methods of communication, *describing,* and *indicating* (Clark, 1996, 2016). Clark (2016, p. 325) defines depictions as “physical scenes that people stage for others to use in imagining the scenes depicted”. Whereas depiction is a broader phenomenon of iconic meaning representation, during enactment, signers/speakers map a referent onto their body “on a real-world scale” (Cormier et al., 2013, p. 370), constructing this referent’s actions and/or dialogs (see Beukeleers and Vermeerbergen, 2022 for a historical overview of the terminology). Imagine the difference in options for depicting someone who is walking. One option would be using your hand, with index and middle finger pointing downward and moving alternatingly for depicting on a miniature scale with a gesture or sign. Another option would be moving your arms as if walking, where the act of walking is mapped onto the signer’s/speaker’s body, and therefore considered an enactment.

Not only enactment itself but also the projection and framing of enactments in a stretch of discourse is multimodal. There is a range of lexical cues that have been described for English to open slots for enactments, such as verbal indices (*like this*), quotatives (*say, ask*), particles (*just, kind of, like*), or other syntactic projections (Tannen, 1986; Hsu, 2021, p. 105; Hsu et al., 2021). Investigations of enactments in British and Australian Sign Language (BSL, Auslan) have shown that signers typically frame their enactments with lexical noun phrases and/or pointing actions, which index the subsequently enacted referent (Cormier et al., 2013; Ferrara and Johnston, 2014). In spoken interaction, prosodic shifts, such as the use of lower, or higher pitch or a louder voice and changes in vowel quality, often proceed enactments (Günthner, 1997). Additionally, in both signed and spoken conversations, shifts in eye gaze (Engberg-Pedersen, 1993; Maury-Rouan, 2011; Thompson and Suzuki, 2014) as well as body orientation (Cantarutti, 2020) or head position (Maury-Rouan, 2011) are indicators that an enactment will follow. Specific gaze patterns have been observed, for example, depending on whether the enacted scene is dialogic or not (Pfeiffer and Weiss, 2022). Furthermore, qualities of the enactment, such as specific prosody or facial expressions, are sometimes already used in the discourse leading up to the enactment and therefore contribute to its framing through a spillover effect (Cantarutti, 2020).

A body of work shows that enactment and depiction practices form a very common way of representing information (Clark, 2016; Ferrara and Hodge, 2018; Hsu et al., 2021; Beukeleers and Vermeerbergen, 2022), both in signed and spoken languages. In narratives, for example, enactment is frequently used to show how something happened or what was said and how. Viewpoints and roles can alternate quickly, shifting back and forth from show to tell. Moreover, viewpoints can be combined through body partitioning, as introduced by Dudis (2004), including or excluding the narrator. Signers/speakers thus often shift back and forth between a narrator’s viewpoint and a character’s viewpoint, to vividly depict an action or discourse situation from a character’s viewpoint, or they combine narration with enactment.

More generally, enactments are always layered and constructed since they are the result of an interactant creatively and selectively “replaying” (Goffman, 1974) activities and (linguistic) actions. Therefore, according to Besnier (1993, p. 161) they are, on the one hand, “the representation of linguistic actions” and, on the other hand, “commentaries about these actions.” This makes enactments a useful tool for expressing stances in interaction (Niemelä, 2010; Cantarutti, 2020). For instance, in the case of music instruction, sonic qualities or movements for instrument manipulation can be depicted as a part of the evaluation of performances and subsequent instruction for musicians by instructors (Meissl et al., 2022). When signers/speakers express a stance during enactment, they create a complex stance-stacking act (Andries et al., 2022; Janzen, 2022). They not only express their stance as a narrator in the here and now but also stack this upon the inherent stance of evidentiality from reporting the speech or action (Shaffer, 2012). Moreover, enactment sequences may report the stances of the enacted characters the signer/speaker depicts during a past event or discourse situation (Debras, 2015). Connected to the expression of a third party’s stance, Debras (2015) has suggested extending Du Bois’ triangle to a tetrad, where another stance subject is added.

Because of the inherently layered nature of enactments and the potential for enactments to express polyphonic (Günthner, 1999) stances, they are a suitable device for mocking. As Debras (2015) states, speakers can distance themselves from an absent subject’s stance by enacting it. As such, multiple stances can be expressed through enactment simultaneously, e.g., by enacting a referent in an exaggerated or stereotypical way to mock them by means of a caricature of a social category attributed to the referent. Fischer and Simon (2016), for example, describe how DGS (German Sign Language) signers use exaggerated mouth gestures in negatively evaluative enactments of hearing people. However, as will be shown in Section 4, mocking can be achieved through a range of different mechanisms next to parodistic imitations. Following the recent studies of Cantarutti (2020) and Mandel (2022), we scrutinize the potential of enactments as a resource for layered stance expressions.

### 2.4 Research aims

The aims of the current contribution are to shed light on (1) how enactments are used for mocking; (2) which layers of stance-taking (on all components of the Stance Triangle) are at play; and (3) how these enactments (and layers of stance) are multimodally constructed.

To feed into the overarching research question on how enactment is used for mocking and which layers are construed multimodally as a result, several smaller-scale questions guided the analysis. For each example, we will scrutinize (a) who or what is enacted, (b) what the stance object is, and (c) who is the target of the mocking. Building on these questions, Section 4.2 discusses (d) what position the stance subjects assume and Section 4.3 focuses on (e) how stance subjects negotiate alignment. These questions allow for teasing apart layers on each component of the Stance Triangle.

## 3 Data and method

To address our research questions, we analyzed the phenomenon of mocking enactments in different interactional settings and several languages, including three spoken and one signed language.

For the **music instruction setting**, two video corpora were consulted. First of all, we used approximately 27 h of wind and brass orchestra rehearsals in Flanders, Belgium (Schrooten and Feyaerts, 2020). Five different conductors were filmed during three rehearsals each with their respective ensemble, and the language of interaction is Dutch. The second corpus contains approximately 8 h of recordings from three chamber music coaching sessions (MuTh, 2021), in which one string quartet worked with three different coaches on consecutive days. The first two sessions are in German and the third in English.

Concerning data on **spontaneous conversations between friends**, two video corpora were used, amounting to 9 h of data in total. Both corpora consist of Dutch triadic interactions. The first corpus (Brône and Oben, 2015) includes eight recordings of spontaneous interactions and eight recordings of brainstorming sessions,^2^ in which participants wear head-mounted eye trackers (for an elaborate description of the corpus and setup, see Jehoul, 2019). The second corpus (de Vries et al., n.d.) contains 12 recordings of spontaneous triadic interactions between friends in a coffee bar and includes 3 camera perspectives on the faces and upper body of each participant. Participants received no instructions for these conversations.

The **narrative data in Flemish Sign Language** are dyadic conversations taken from the corpus Flemish Sign Language (Van Herreweghe et al., 2015). The data used for this project consist of 5 h of dyadic conversations of 34 signers. Two types of conversations are used: (1) free conversations without a moderator being present, during which the participants could talk about whatever they liked, and (2) guided conversations about topical past events. During those guided conversations, the participants were shown a photograph of a historical event such as 9/11, the 2004 Indian Ocean earthquake and tsunami, and the conviction of the Belgian pedophile Marc Dutroux. The signers were invited to talk about what they remember of this event, where they were when they learned what had happened, and how they experienced it.

We first scanned the different video corpora for sequences with mocking enactments. As is the case with many phenomena connected to humor or non-seriousness in interaction (Gibbs, 2000, p. 12), it is not always evident to draw strict lines between what counts as ‘mocking enactment’ or not. To balance out individual subjective judgments, we maintained an inter-coder negotiation process throughout the data segmentation and analysis period. As a baseline, approximately 10 cases per data set were discussed in groups of three. For the data in VGT, two deaf signers were consulted for their judgment. For the data in spoken languages, the three authors discussed the case selection, and throughout the analysis process, cases of doubt were double-checked within this team. These discussions facilitated a selection of approximately 30 cases per setting that form the basis for the current analyses. Tendencies and patterns that surfaced in these cases will be discussed on the basis of five examples in the following sections.

## 4 Analysis

### 4.1 Evaluation

In this first part of the analysis, we turn to the music instruction setting and discuss how mocking enactments can be used for purposes of evaluation during instruction. The two examples illustrate how the use of absurd imagery by a conductor (Example 1) as well as a self-deprecating enactment by the coach in a master class (Example 2) can contribute to evaluations of stance objects on different layers. For each example, we will discuss who or what is enacted, what the stance object is, and who the target of the mocking is, to then elaborate on how this feeds into the evaluation process that is central to the setting of music instruction.

As a first example, we will look at an extract taken from a recording of an orchestra rehearsal (see Supplementary material S1).^3^ In this setting, there is a characteristic temporal organization of alternating sequences of play and sequences in which play is interrupted and the conductor offers feedback and instructions (cf. Stoeckl and Messner, 2021). Example 1 marks a typical instructional sequence, in which a contrast between an undesired and desired performance is expressed (Weeks, 1996; Meissl et al., 2022). We will discuss the example in two parts, initially focusing on lines 01–08 where the first mocking enactment is staged, and then turn to the rest of the sequence in lines 09–21 with subsequent enactments.

The conductor interrupts a playing sequence after the first few notes and addresses the flute section (lines 01–02). With a raised voice, the conductor utters a rhetorical question, asking the musicians what scares them off about their first note—a B-flat—and thereby localizes the problem in their previous performance: their hesitancy and a delayed onset of the first note. This question is quite affectively loaded given the prosody and the conductor’s tense gesture with clenched fists in line 03. He reformulates his question and leans slightly backward (line 04), distancing himself physically from the musicians. Lines 01–04 therefore mark the first negative evaluation of the preceding performance as a stance object.

**Figure 2:**
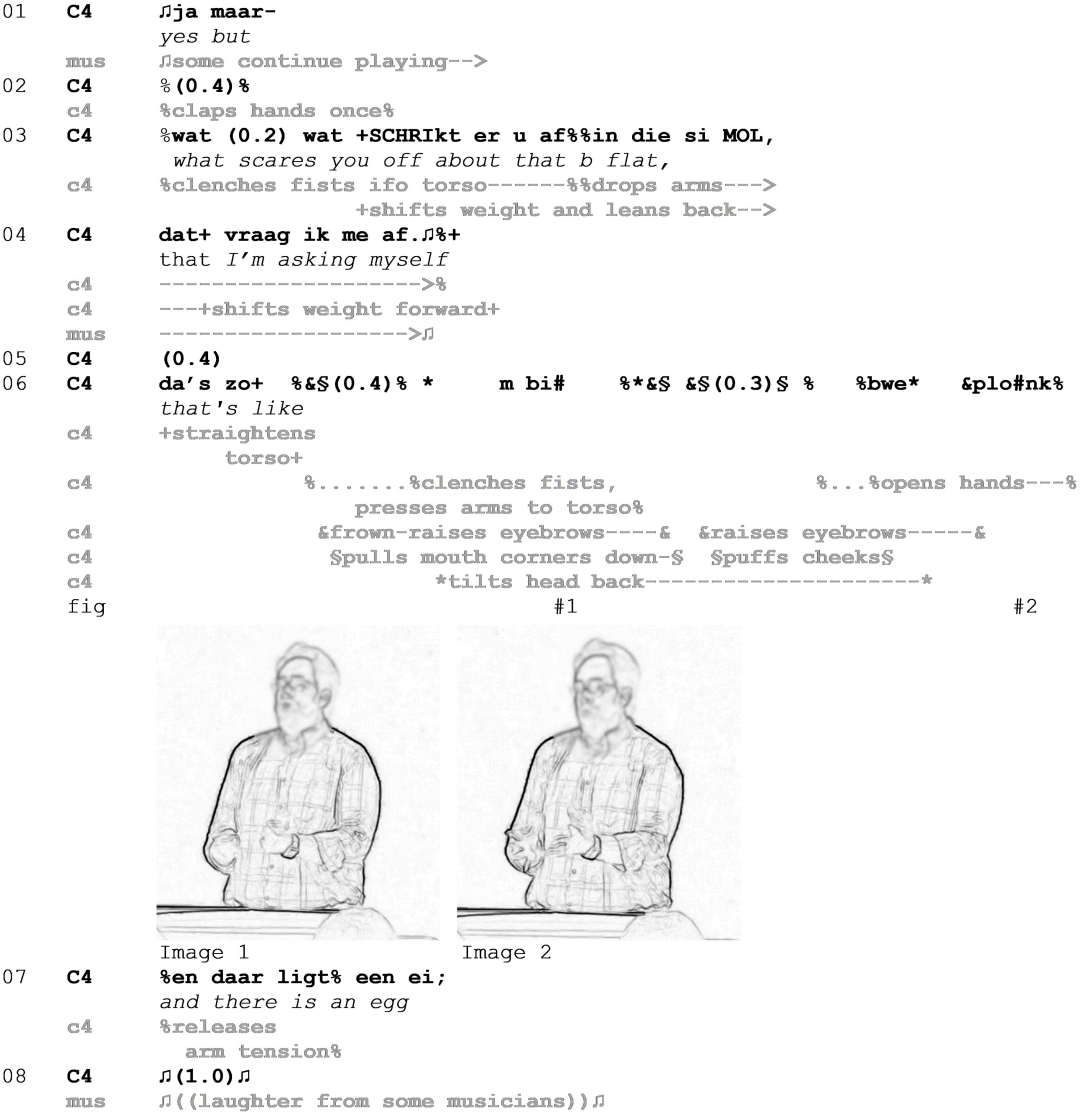
Example 1.

After a short pause, in line 06, the conductor sets the stage for the mocking enactment by uttering “da’s zo” (*that’s like*), referring to the (performance of the) first note. Typical for this setting, the formulation he uses projects information about the undesired performance. The opened syntactical slot is filled by an enactment, which can be split into two parts, the first marked by a sense of ‘tension’ and the second by ‘release’. During the first phase, the conductor clenches his fists in front of his body with his arms angled and pressed tightly toward the torso. He frown-raises his eyebrows (Nota et al., 2021) and pulls the corners of his mouth down with tense closed lips. Next, he tilts his head back and looks up. This very tense bodily display is accompanied by two vocalized sounds with a very pressed and tense quality—first a restricted “m” and then a slightly louder “bi” (Figure 2 - Image 1). The second phase of the enactment is initiated with an eyebrow raise, a short flash of puffed cheeks, and subsequently the conductor opens both hands to a cup shape, as if holding an object. This goes together with a sound such as “bwe” and after the hands are already opened, another “plonk” during which the conductor looks at the orchestra (Figure 2 - Image 2). The interplay of visual resources and the sounds with a ‘plopping’ quality results in an expression of tension release. The associations that this complex display may invoke, namely, a chicken laying an egg, are immediately verbalized in line 07. When producing this utterance, the conductor also loosens tension in his shoulders and arms, still contributing to the overall impression of ‘tension release.’

We therefore see, that in the enactment (line 06) and subsequent description (line 07) of the undesired performance, the conductor is referring to the musicians’ performance on the interactional base layer. However, he does so through the addition of absurd imagery on a pretense layer, comparing the onset of a note with laying an egg. Put into perspective with the Stance Triangle, another stance object is introduced: A chicken laying an egg, attached to which comes a whole set of associations with chickens and their behavior. Retrospectively, that semantic domain also fits the feeling of “being scared off,” which the conductor attributes to the musicians in line 03. The chicken laying an egg is mapped onto the conductor’s body, who is also an evaluating stance subject. Through the use of this enactment, the conductor evaluates the imagery of a chicken laying an egg as negative, which in turn serves as the negative evaluation of the musicians’ performance. This layering results in a mockery of the musicians’ previous performance of the first note. During a 1.0-s pause in line 08, there is audible laughter from some members of the orchestra, showing that the enactment has caused amusement.

**Figure 3:**
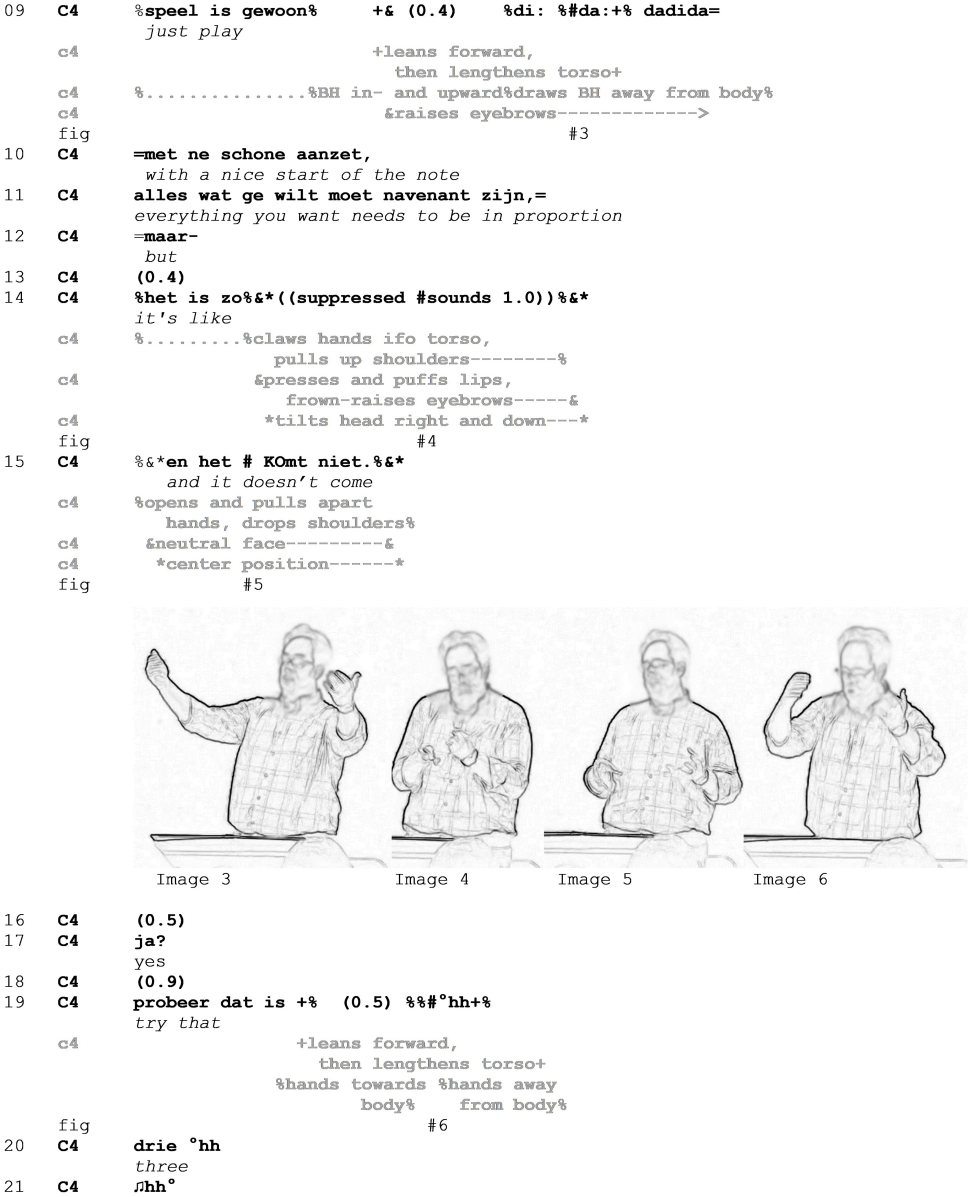
Example 1.

Now, we will turn to lines 09–21 of the example to see how the conductors’ evaluation and instruction progress. In lines 09–11, the conductor depicts and describes the desired performance, highlighting the initiation of the first note (Figure 3 - Image 3). The contrastive conjunction “but” (line 12) projects the return of focus to the undesired performance, which is continued in line 14 with a slot opener for another mocking enactment. This shares a range of qualities with the first part of the previous egg-laying enactment in line 06. The conductor clenches his hands in a claw handshape, presses his arms to his torso as before, and pulls up his shoulders. His head is tilted right and pulled down, resulting in a compression of his torso. The tension is also visible in his face, with a frown-raise and pressed as well as puffed lips. Through these tense lips, some suppressed sounds are audible. Thus, the notion of tension and effort is reinvoked (Figure 3 - Image 4). The release, however, only follows with the utterance “and it does not come” (line 15), a construction that expresses both the desired result—the note coming out—and the fact that this had not happened in the previous performance (Figure 3 - Image 5). Thereby, the conductor highlights the incongruence between his expectations and the reality of the musicians’ actions. What follows this extract is the repetition of the desired performance qualities (Figure 3 - Image 6) and the initiation of another playing sequence in line 21.

Summing up, Example 1 features two mocking enactments, in which a conductor offers parodistic imitations of the musicians’ previous performance, a common tool in this setting to highlight undesired qualities of performances (Poggi, 2015). We have shown that this results in the layering of the stance object and evaluation through exaggeration and absurd imagery.

While such parodies of co-present participants are probably the most common format in which mocking enactments appear in the setting of music instruction, we will now turn to an instance in which a mocking enactment is used as a form of self-deprecation based on invoking an absurd scenario (see Supplementary material S2). A different kind of evaluative layering is brought about by coach Stefan Gottfried (henceforth SG) in a string quartet master class. The ensemble members are—seated from left to right—Marie-Therese Schwöllinger (MTS), Alexandra Moser (AM), Anuschka Cidlinsky (AC), and Oscar Hagen (OH).

**Figure 4:**
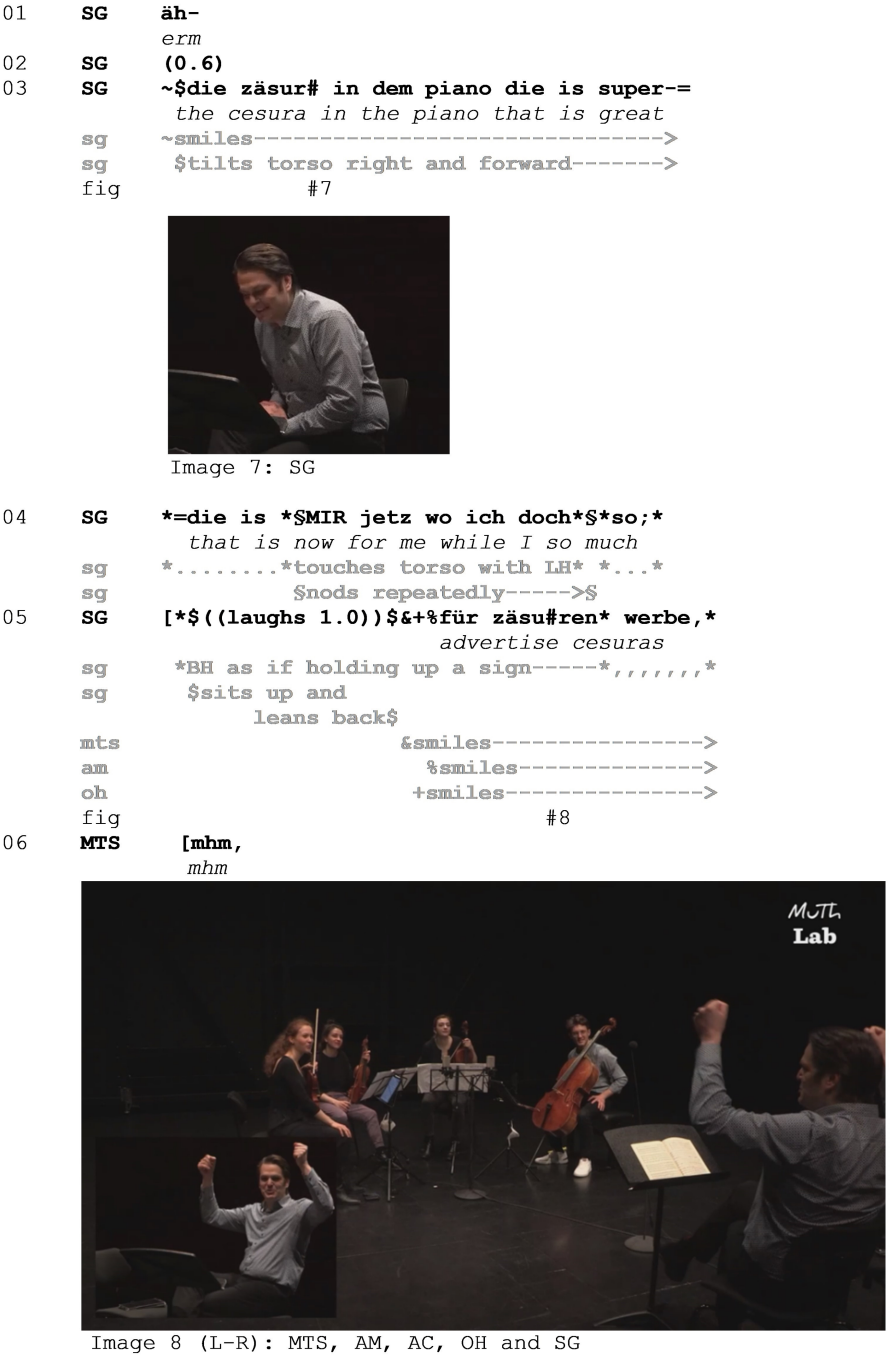
Example 2.

**Figure 5:**
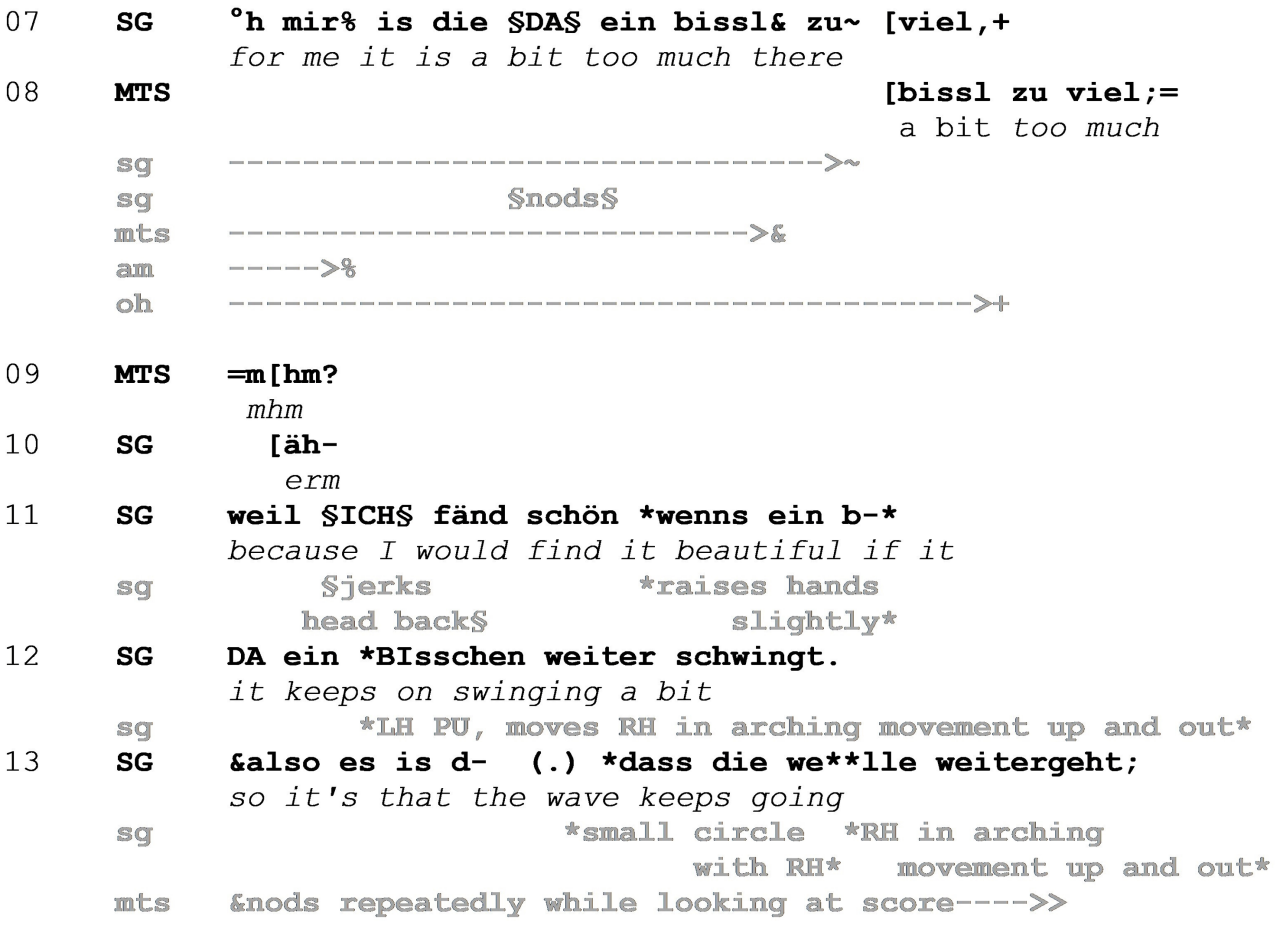
Example 2.

During the session preceding Example 2, coach SG has suggested to use *cesuras* as expressive elements. A cesura is an interruption between notes that can function in a way similar to a comma in a spoken sentence, resulting in a pause between two phrases. After several sequences of play and interruptions in between, SG returns to the topic of cesuras in Example 2, referring to a specific instantiation in the piece (line 03). He evaluates the realization of the cesura positively as ‘great’, smiles, and tilts his torso to the right and slightly forward (Figure 4 - Image 7), which can be interpreted as a hedging device projecting the subsequent modification of his evaluation in line 04. SG utters “die is mir jetzt” (*that is now for me*), stressing “mir” (*for me*) and touching his chest as a deictic gesture toward himself. Before this sentence is finished, however, an adverbial contrastive subclause is inserted, which also contains the mocking enactment. SG says “wo ich doch so” (*while I am so much*) (line 04) and laughs for about 1.0 s (line 05), during which he lifts his two hands up as if holding up a sign, mapping the action onto his body. While holding his hands in the same way above head height and smiling (Figure 4 - Image 8), he utters “für zäsuren werbe” (*advertizing cesuras*), completing the subclause that as a whole translates roughly to ‘while I am advertising cesuras so much’ and therefore refers back to the instructions he has given before this extract. Completing the main clause that was started in line 04, SG finishes his evaluation of the performed cesura in line 07 (Figure 5) by saying that it was a bit too much for him there. Through the mocking enactment, in which SG frames his repeated instructions as an advertisement for an artistic means of expression, he positions his previous instructions as the stance object and himself as the target of the mockery. SG marks his own previous actions as laughable, which is reciprocated by the musicians through smiling (lines 05–08).

The stance object therefore evolves from the musicians’ realization of the cesura to the instructions that SG has previously given. Note that in contrast to the mocking enactments in Example 1, the enactment by SG here does not draw on means of exaggeration in its multimodal orchestration. It is rather the absurdity that lies in the analogy through which advertisement and holding up a sign for promotion is mapped onto the action of instructing in a musical setting. Through this absurd analogy, another level of mocking is achieved which lies in the incongruence between his previous and current stance toward cesuras. This is highlighted by the use of the inserted contrastive subclause (lines 04–05) in which the enactment is embedded. While promoting cesuras before, and therefore influencing the musicians’ performance now up for evaluation, SG deems the cesura too much at that specific point (line 06). He acknowledges that incongruence through self-deprecation which allows him to take responsibility for the musicians’ performance of said cesura and results in smiles from the members of the ensemble.

In line 07, the first violinist (MTS) repeats the last words of SG’s ongoing turn in overlap, showing uptake of that evaluation, and gives a confirming ‘mhm’ in line 09. Following that, SG goes on to explain, what the desired rendition of the fragment in question would be (lines 11–13), in contrast to the unwanted interruption through the cesura. MTS nods continuously in line 11 while looking at her score, again signaling alignment.

Examples 1 and 2 have shown that evaluative stance-taking in instruction can be layered in different ways. In Example 1, the conductor performs a mocking enactment of the musicians’ performance which leads to evaluations on two layers: one of the actual previous performance on the base layer and one of the image of a chicken laying an egg and connected associations on a pretense layer. The overarching stance object, which is the musicians’ performance, overlaps with the target of the mockery, namely, the musicians. In Example 2, a different kind of stance-stacking is achieved through self-deprecation by the coach. Embedded into the hedged negative evaluation of the previous performance, there is a negative, mocking stance toward SG’s own instructions on another layer. The examples illustrate the constant dynamic evolution of stance objects, which Iwasaki (2022, pp. 7–10) has highlighted previously in terms of their temporal unfolding. In addition to this sequential development, we can see that mocking enactments coincide with a layering of objects and their evaluation at one moment in time, through the combination of serious and non-serious layers.

While in Examples 1 and 2, there is mostly congruency between who/what the stance object is in relation to the target of the mockery, the mocking enactments serve very different functions in terms of positioning of the subject who performs them. This intricate positioning process will be the focus of the next section.

### 4.2 Positioning

In the second part of the analysis, we turn to spontaneous conversations between friends and zoom in on the process of positioning. We scrutinize how the target of the mockery is positioned, as well as how the participants distance themselves from the stance object and the target of the mockery. Using two examples, we show that, similar to evaluation, positioning is a layered endeavor. In both Example 3 and 4, participants invoke other viewpoints instead of (Example 3) or next to (Example 4) the viewpoint of the target of the mockery. In the first part of the analysis, we discuss who or what is enacted, what the stance object is, and who the target of the mocking is. Additionally, we analyze how participants position themselves.

Consider Example 3 below, taken from the coffee bar corpus (see Supplementary materials S3, S4). In this example, three friends from university (Michael, Noor, and Melanie) are discussing how much they like fresh soup but that it is so expensive to buy in the supermarket ready-made. First, we will examine lines 01–08, including the build-up toward the mocking enactment. Next, lines 09–13, including the mocking enactment, will be discussed.

In lines 01–03, Michael proposes that it is not that much trouble to make soup yourself if you buy an immersion blender. During his proposal, Noor bursts out in laughter (line 04), which is attended to by both Michael and Melanie. Melanie then explicates the laughable (lines 06–08) and mocks Michael’s proposal, arguing that it is a ridiculous idea to spend so much money on “such a fucking thing.” A discussion follows about the pros and cons of making your own soup (the omitted lines).

**Figure 6:**
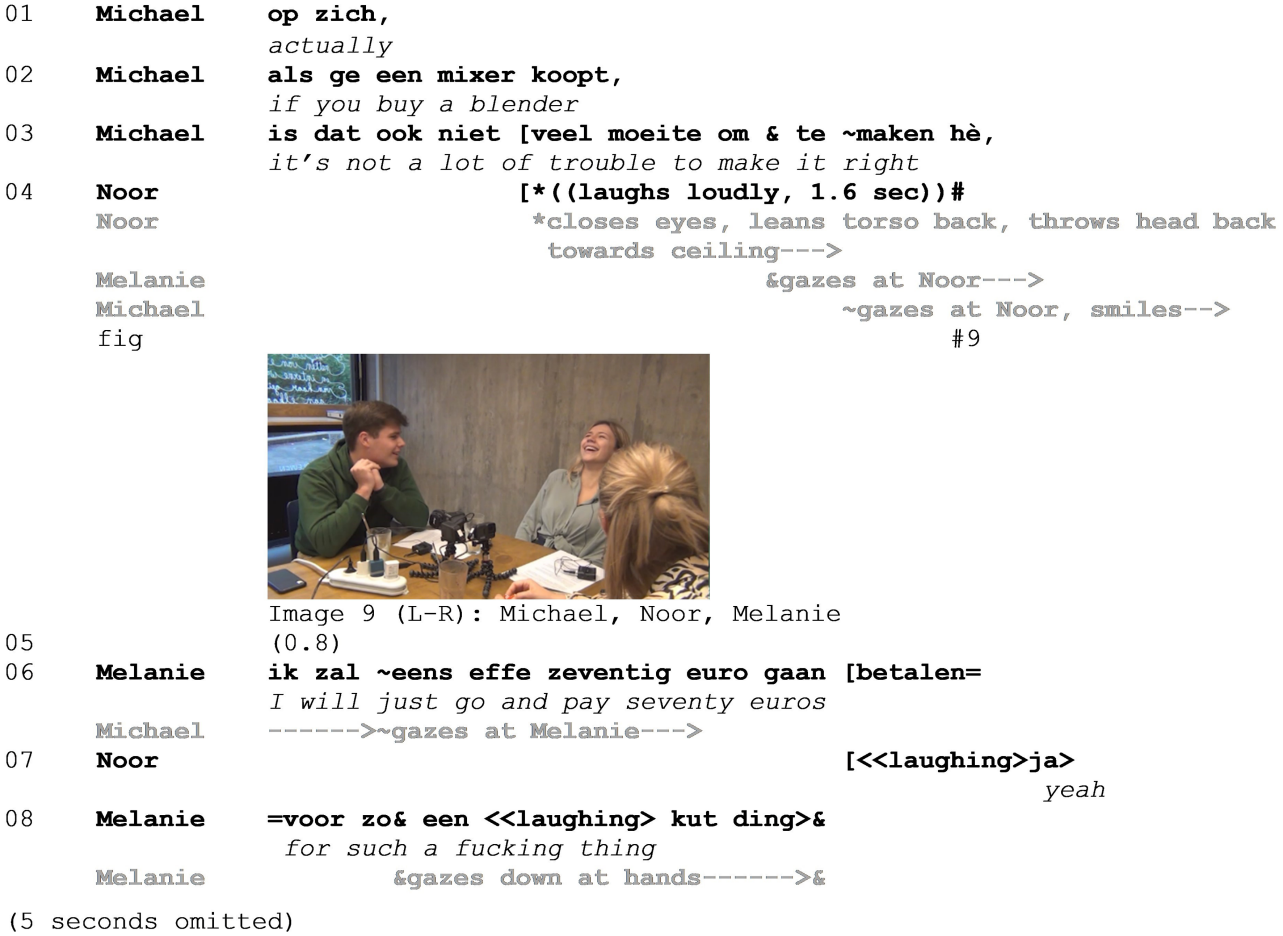
Example 3.

In line 04, when Noor bursts out in laughter (Figure 6 - Image 9), she produces the first negative evaluation, evaluating Michael’s proposal to “buy your own blender” as laughable, and clearly distances herself from Michael’s stance. At the same time, this opens the floor for Melanie to explicate the laughable and produce the first mocking utterance, which is not yet an enactment. Shifting pronouns from a generic “ge” (*you*) to the specific hypothetical of Melanie herself (“ik,” *I*), she stages a layered evaluation of Michael’s proposal. By pretending to go along with him and “just go and pay 70 euros for such a fucking thing,” the contrast between two scenarios is invoked, in which paying 70 euros for a blender is an excellent idea versus a terrible idea, resulting in a stacked stance. The layered evaluation serves to position Michael’s proposal as absurd. Furthermore, Melanie’s use of the first-person pronoun “I,” denoting her hypothetical self, in combination with her gaze averted to her hands, contributes even more to the distancing with respect to Michael’s proposal (see also Haddington, 2006).

**Figure 7:**
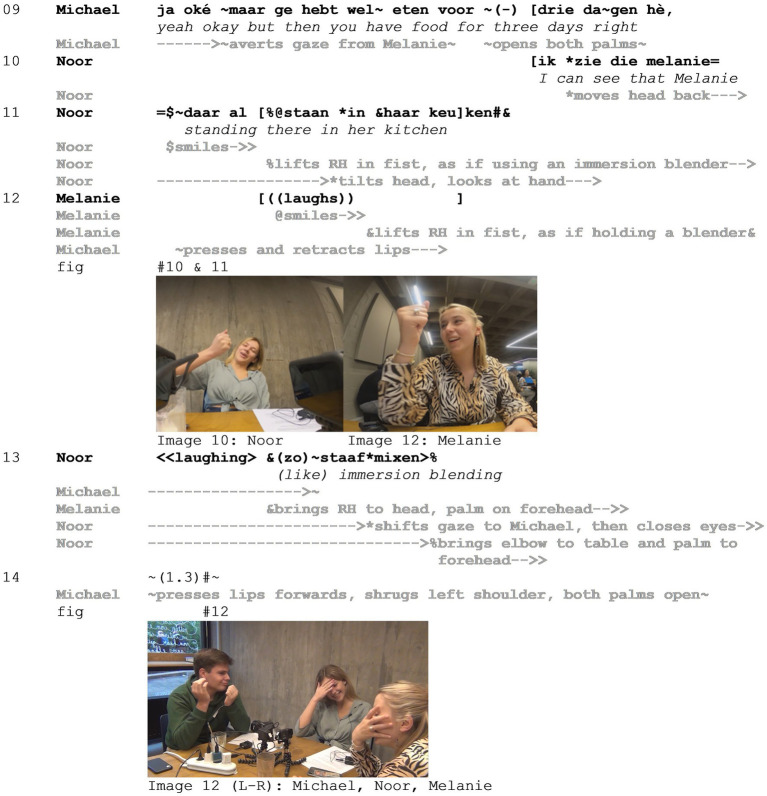
Example 3.

Subsequently, a serious discussion follows concerning the pros and cons of making your own soup with an immersion blender (the omitted seconds). In line 09, Michael argues in favor of making your own soup but is interrupted once more by Noor (line 10), who then produces the mocking enactment and is joined by Melanie (lines 11–13, images 10 and 11). Finally, in the silence that follows, Michael produces a compound shrug (line 14, Figure 7 - Image 12).

Continuing his argumentation (line 09), Michael resists Melanie’s and Noor’s line of thought (averting his gaze from Melanie) and positions his idea as obvious, as indexed by the palm open gesture with both hands (Marrese et al., 2021). In her interrupting turn then, Noor verbally sets up a stage for the hypothetical scenario and the enactment to come (“I can see that Melanie”). Note also the switch in pronouns similar to the first part, from the generic “ge” (*you*) in line 09 to “die Melanie” (*that Melanie*) in line 10. This switch is interesting in two ways. First, it presents a shift from a general proposal to a specific hypothetical scenario, emphasizing Melanie’s role in it. Second, it functions to distance Melanie the addressee from “die Melanie” (*that Melanie*) involved in the fictional scenario that is about to be staged.

In the enactment, Noor uses her right hand to depict using an immersion blender, while visually attending to this action. At this point, it may be noted that the enactment itself is not staged by drawing on exaggeration (as is the case in Example 1 above). Rather, the depiction of this action serves to draw attention to the incongruence between the act of blending soup and Melanie being the one performing the action (much like in Example 2 above). This incongruence gives rise to the positioning of the proposal as absurd, the positioning of Michael as the target by extension, as well as the distancing of Noor and Melanie from the stance object and target (i.e., the stacked stance).

Finally, following their enactments, Melanie and Noor cover their faces with their hands (Ford and Fox, 2010) visually distancing themselves from the scenario and dismissing the idea (Figure 7 - Image 12), while Michael continues to resist and produces a final shrug.

It is interesting to note the incongruence between the character of the enactment and the target of the mockery. Instead of depicting Michael, Noor positions Melanie as the character of the enactment. In isolation, the enactment in lines 10–12 could be interpreted as mocking Melanie. Melanie is positioned as a ‘typical student’ who does not put too much effort into cooking, whereas Michael positions himself as a responsible person who thinks ahead (e.g., about having food for 3 days, line 09). It is especially the misfit of the idea of Melanie blending soup, and the difference between Melanie and Michael, that gives rise to the mockery. However, in light of the preceding turns (lines 01–08), including both Noor and Melanie mocking Michael’s proposal, we argue that the low likelihood of Melanie blending her own soup merely serves to highlight the absurdity of Michael’s proposal, rather than staging Melanie as a target as well. This in turn highlights the notion that positioning with regard to the stance object and target is done locally in the interaction but also draws on and extends to positioning in a wider sociocultural field including membership categories (see Section 5.3).

Another tool that participants have at their disposal to distance themselves from the stance and target at hand, and which emerged prominently in our data set, is by including a viewpoint shift immediately following the mocking enactment. As an illustration, consider Example 4 (see Supplementary material S5). In this extract, three friends (Jilske, Yana, and Nikki) are talking about Lucas, a friend of theirs, who forgot to take his lab glasses to class and then came rushing into Jilske and Nikki’s class to ask whether he could borrow one from them. Immediately preceding the excerpt, all participants are displaying what could be interpreted as *Schadenfreude* on the incident. In lines 01–03, Nikki adds to the anecdote that Lucas did bring his lab coat “in compensation”. In lines 07–10 then, many things happen simultaneously. Nikki produces a mocking enactment, in which she depicts Lucas offering his lab coat “in compensation.” Simultaneously, both Yana and Jilske evaluate the situation as a whole. Finally, in lines 14–15, Nikki adds another enactment, this time from her own viewpoint, mocking their friend once more.

**Figure 8:**
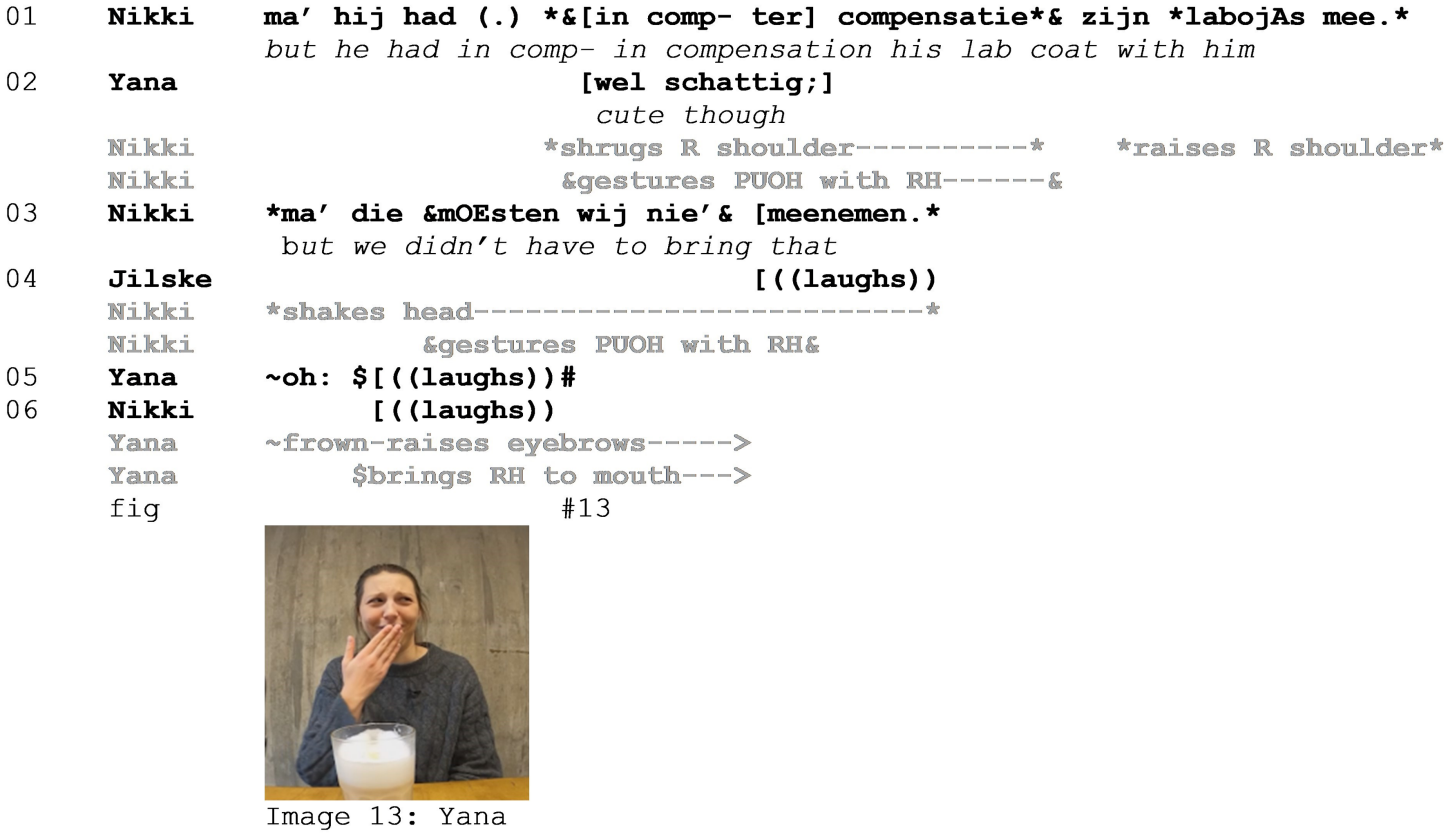
Example 4.

Throughout the example, all participants position Lucas as the target of the mockery. In line 01, Nikki utters the statement that he brought his lab coat “ter compensatie” (*in compensation*), while producing a palm up open hand gesture (PUOH) as if presenting the lab coat, and a shoulder shrug, distancing herself from this action. While uttering “labojAs” (*lab coat*) with emphasis on “coat,” she raises her shoulder again, highlighting the discrepancy between what was expected—to bring glasses—and reality—that he brought a coat. This is exploited further in line 02 when Nikki explains that “they did not need to bring that,” a statement accompanied by another PUOH gesture and a head shake, presenting this as obvious and referencing the failed expectation. Both Yana and Jilske align with the stance at hand by laughing in lines 04–05, and Yana frown-raises and covers her mouth with her right hand (line 06, Figure 8 - Image 13). This gesture can be interpreted as displaying vicarious embarrassment (or *Fremdscham*) on Lucas’ behalf, or self-censorship of a potentially inappropriate response to the event. In all, her response contributes to the evaluation of Lucas’ actions as embarrassing and as such distancing herself.

**Figure 9:**
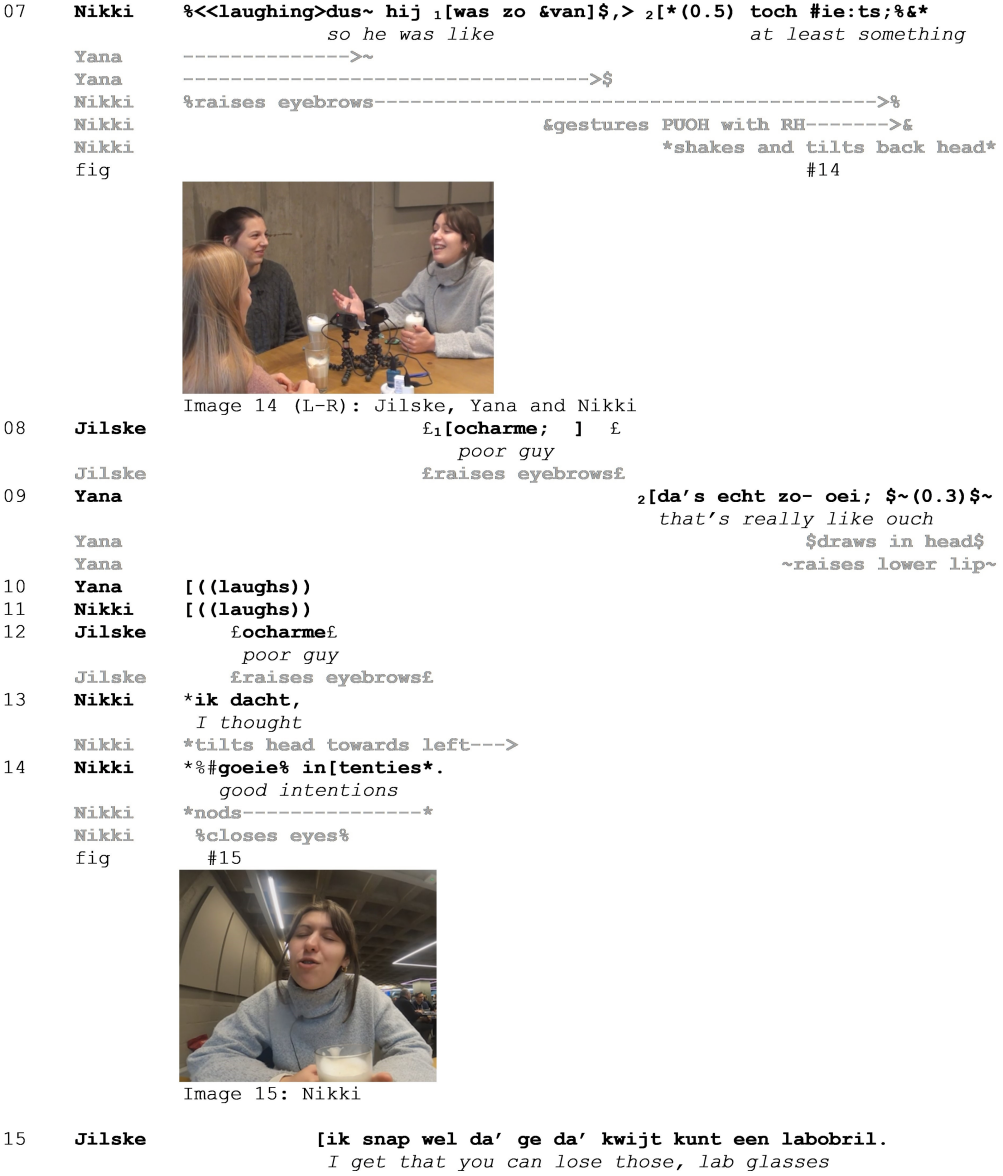
Example 4.

In line 07, Nikki enacts Lucas (“*and he was like, at least something*”), which, by means of the raised eyebrows, PUOH gesture, and head shake and tilt (Figure 9 - Image 14), gives rise to a feeling of desperation, as if pleading for his case. The enactment highlights the inappropriateness of Lucas’ action—the unsuitability of a lab coat to replace lab glasses—resulting in the mocking character. Simultaneously, Yana produces another enactment (line 09). In this enactment, it is unclear who the enacted character is—herself, Lucas, or a more general audience. What emerges can be described as a doing evaluating, in which not so much the positioning of a specific stance subject is relevant, but rather the evaluation of the situation as embarrassing, or a situation in which one would say “ouch.” Nikki then adds another viewpoint (next to the viewpoint of the target of the mockery from line 07, and the viewpoint of a generic evaluator in line 09) in an enactment of her own (mocking) stance in response to the events. She positions herself as a calm, potentially authoritative figure, praising his effort (“goeie intenties,” *good intentions*) while tilting her head, closing her eyes, and nodding (Figure 9 - Image 15). This layered evaluation presents a contrast with the first enactment of the desperate target of the mockery and even more directly distances the speaker from the target.

Jilske, finally, somewhat resists the mockery and affiliates with Lucas’ position (in lines 08 and 13, saying “poor guy”). Indeed, after the extract, she continues with a defense of Lucas’ actions, saying that she understands how you could lose your glasses. In Section 4.3 below, we will discuss how participants sometimes partially align with separate layers of the mocking stance.

In sum, this section described how participants (1) position the target of the mockery and (2) distance themselves from the stance object and target of the mockery. The layered evaluation (as presented in Section 4.1) can use absurd scenarios including analogy and imagery, as well as exploit the contrast between some expectations and reality, often drawing on broader membership categories and stereotypes. Together, this results in a layered positioning in which (a) the character of the enactment, (b) the stance object, (c) the target of the mockery, and (d) the interlocutors in the interaction are positioned. With regard to the role of different viewpoints in positioning, we saw that participants draw on layered viewpoints: Next to enacting the target of the mockery, participants can enact themselves to include their own viewpoint and position. In some cases, the target of the mockery is not the character of any enactment in the interaction at hand.

### 4.3 Alignment

In this section, we focus on the layering of alignment. As discussed in the previous examples, during mocking enactments, interactants construct a layering of both evaluations as well as positioning. Since multiple objects and multiple subjects are involved in the stance act, the negotiation of alignment can gain complexity. Not only can all present interactants (dis)align with each other and with the enacted character, but they can also do this for multiple evaluative layers toward the object separately (e.g., only align with the non-seriousness but not with the evaluation of the object).

In this section, we look at one longer sequence containing multiple mocking enactments that will illustrate the layering of alignment and the complexity of its negotiation.

**Figure 10:**
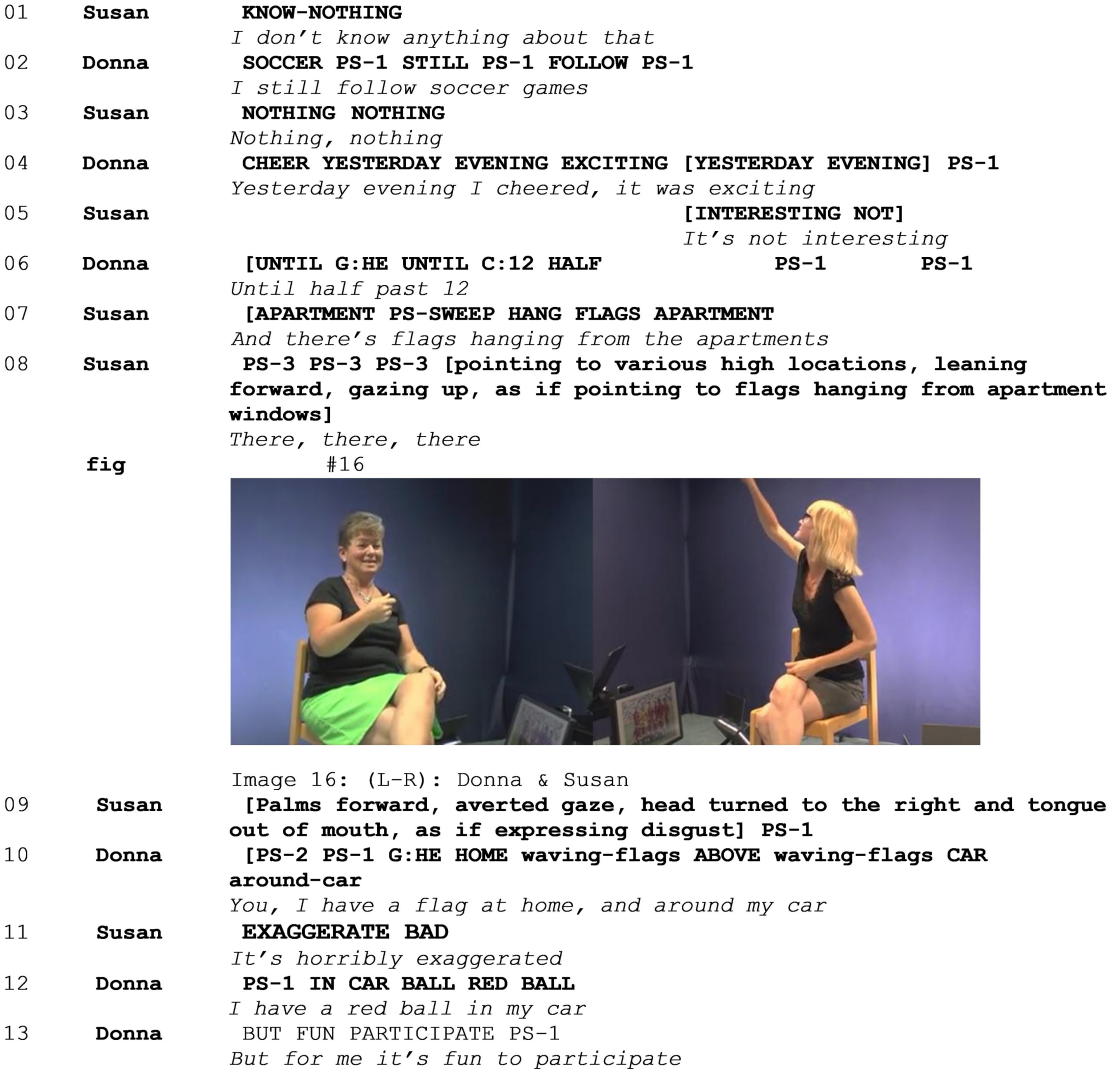
Example 5.

In Example 5 (see Supplementary materials S6, S7), two signers (Susan and Donna) are discussing the ways people cheer for games of the Belgian national soccer team. When confronted with this topic in the elicitation task, Susan immediately states that she does not know anything about soccer and produces an away gesture, waving the topic away (Bressem and Müller, 2014), and expressing her stance on the topic both lexically and non-lexically. Donna, however, expresses her enthusiasm about the sport, smiling, and nodding while she states that she saw a game the day before. Subsequently, Susan states that this is not an interesting topic. The two immediately express disaligning stances on the topic in overlapping turns (lines 4–7), both epistemically and affectively. Then, Susan produces a mocking enactment: In line 08, she enacts herself looking at Belgian flags hanging out of the windows of apartment blocks and pointing at these flags (Figure 10 - Image 16). This enactment is followed by a second one, in which Susan makes a palm forward gesture, averts her gaze, turns her head to the right, and sticks her tongue out of her mouth, expressing her disgust. In this example, the first mocking enactment is viewpointed from the signer’s perspective. The character of the enactment is the signer herself in the past, and the target, people who hang out Belgian flags during soccer games, is only implied. While enacting herself looking at apartment windows, feeling disgusted when she sees these flags hanging out of windows, she not only positions herself, evaluating this object. Rather, within the context of this interaction, she also positions people who hang a flag out of their window as the target of ridicule. As discussed in Section 4.2, there is an incongruence between the character of the enactment and the target of the mockery. During these two mocking enactments, Donna smiles and, in overlap with the second enactment, points at herself. She signs that she herself hangs the Belgian flag out of her window during soccer games. In line 10, she continues by saying that she also decorates her car and that she likes doing that. As such, she makes clear that she is a member of the group of people that constitutes the target of the mockery in Susan’s enactment. While she does that, Susan shakes her head and gasps, expressing her negative stance. Susan continues in line 11, saying that “it’s horribly exaggerated.” In this example, we can observe how the two signers do not align with each other, engaging in a heightened multimodal stanced discussion, combining a range of resources to express their disalignment in overlapping turns, in the run up to a mocking enactment. Moreover, it becomes clear that Donna is—possibly by accident—not only a subject who can align with the mocking stance but also the target of the mockery. Subsequently, the two elaborate on the topic, engaging in a 1-min discussion on the topic of using flags and decorations to cheer during soccer games, overtly expressing their strong disalignment (lines omitted in the transcript).

**Figure 11:**
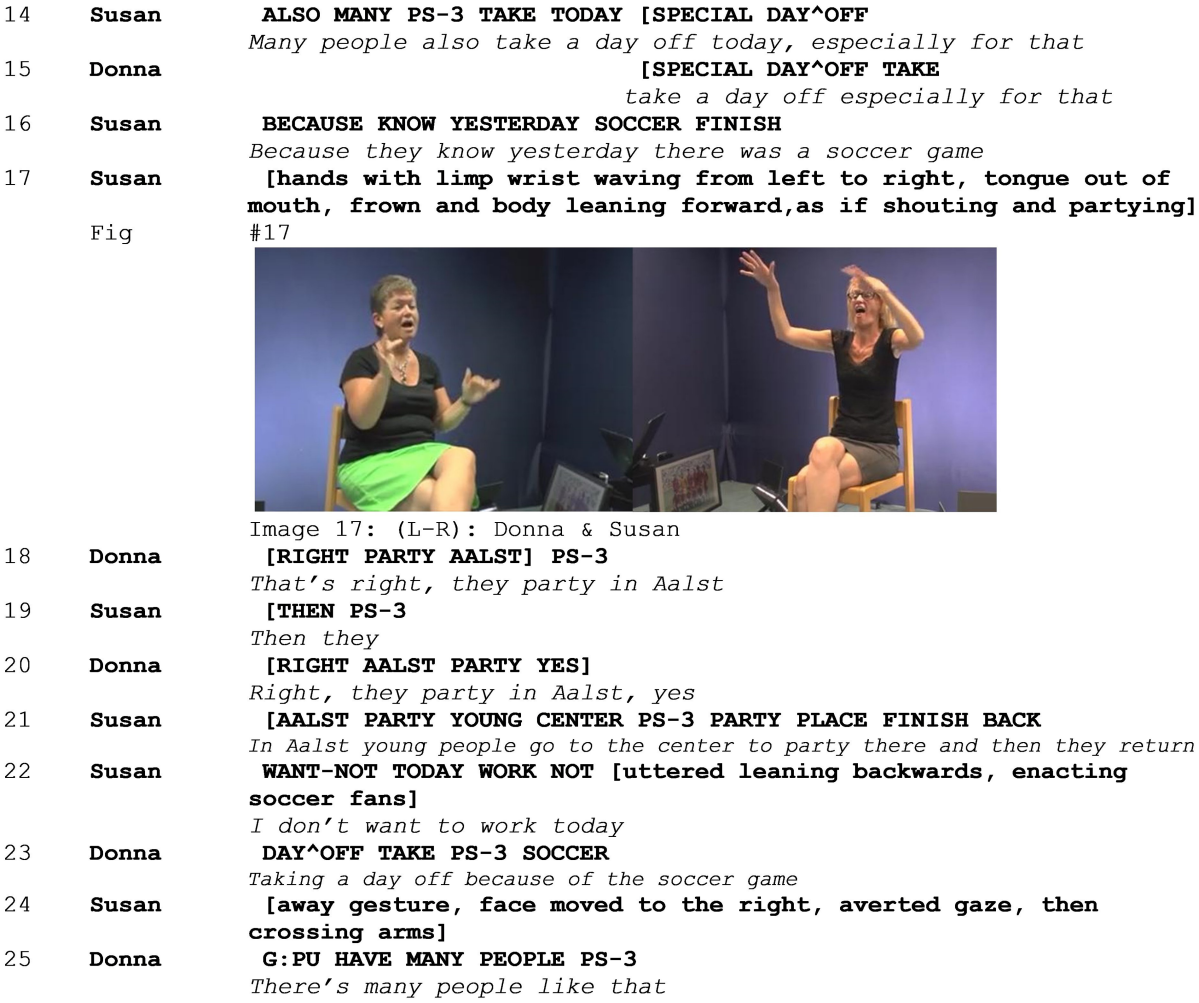
Example 5.

After engaging in a discussion, Susan signs that some people take a day off to go out and party the day after a soccer game, frowning, opening her eyes wide, and moving her body forward. Donna leans back, smiles, and nods, agreeing that this is true. With this enactment, Susan shifts her viewpoint, enacting the target of the mockery, distancing herself from the stance and target, as discussed in Section 4.2. Then, Susan produces another mocking enactment in line 16, enacting people who go out to party in the Belgian city of Aalst and, waving her hands from left to right, sticking her tongue out of her mouth, frowning, and leaning forward, as if shouting and partying (Figure 11 - Image 17). Donna aligns, smiling and saying that is true. Susan elaborates on this, clarifying that she is talking about young people who go out to party in the city center. She continues this enactment, showing how these people say that they do not want to work the next day and take a day off. With this second mocking enactment sequence, the target of the mockery narrows down to a more specific group of people. With the mocking enactments in lines 17 and 22, Susan evaluates the object of people who go out to party in Aalst and do not work in a layered manner, creating a stacked stance expression: The enactment serves as an evidential, presenting what she knows about these people, while negatively evaluating them through enacting their behavior in a non-serious, exaggerated manner. As such, in contrast to the previous enactment, she reduces the scope of the group that constitutes the target of the mockery, excluding Donna. Consequently, this mocking enactment sequence serves as a mitigation strategy with regard to the previous mocking enactment. While during the first enactment, the behavior of Donna was negatively evaluated, during this enactment, there is room for her to position herself toward an external target, allowing her to align with her interactant. Thus, the mocking enactment in line 17 constitutes a part of the negotiation of alignment on the topic as a whole and specifically with regard to the previous mocking enactment where one of the interlocutors was a member of the target group. However, in line 20, Donna again expresses that she knows people do take days off after going out to party, but she does not express her alignment on the mocking stance. While Donna aligns with Susan on both the epistemic layer and the non-serious layer of the mocking enactment with smiles, nods, and a lexical expression recognizing that the statement in itself is true, they do not reach alignment on the evaluation. As such, they only partially align on this stacked stance.

It appears, therefore, that the layering of the stance acts has implications for the expression of alignment. When interactants layer the evaluation and positioning in the stance act, they can also only partially align with these complex stances. If so, interactants seem to be more likely to elaborate on these stance acts. This layering of alignment relates to Clark’s (1996) distinction between imagination and appreciation: “When there are two layers, the primary participants are to imagine the actions in layer 2, and appreciate the actions in layer 1” (Clark, 1996, p. 360). It is very well possible that interactants do imagine the enacted scene their interlocutor depicts but do not appreciate their actions on the base layer in the interaction. Indeed, our analysis confirms the findings of Holt (2016, p. 101) stating that layering of playful and serious stances presents the recipient with options in terms of responding to these playful first turns. They can align with playful aspects, with serious elements, or both.

When we consider these aspects of alignment with the presence of multiple subjects, layered stances, and possibly multiple objects, a complex negotiation may arise. As Iwasaki (2022) states, alignment or disalignment does not constitute a binary contrast but a fluid spectrum. The expression of a stance opens an opportunity for negotiation over the course of an interaction. However, we argue that on top of that, this spectrum of alignment and the possibility of partial alignment also exist in specific moments within the interaction.

Moreover, it becomes apparent how stance subjects, objects, and targets may overlap and be intertwined, and how this may evolve over the course of an interaction and implicate the negotiation of alignment. During the first mocking enactment, Susan supposed Donna to be a subject she could align with regarding the object of “putting flags out of your window.” However, it turned out that her interlocutor was not only another subject but also the object of the stance. The signers did not align, until Donna expressed her alignment by acknowledging the epistemic stance and the non-serious stance, but leaving her (negative) evaluation unexpressed.

Summing up, mocking enactments are complex multilayered stance acts that also entail a layering in expressing alignment, whereby interactants can align, disalign, or partially align. In the context of storytelling in interactions, it seems like alignment on every layer is desirable, if not necessary, for the progress of the interaction (Stivers, 2008; Peräkylä et al., 2015).

## 5 Summary and discussion

The aim of this study was threefold: First, we aimed to shed light on how enactments are used for mocking. Second, we asked which layers of stance-taking (on all components of the Stance Triangle) are at play. Third, we scrutinized how these enactments (and layers of stance) are multimodally constructed. In what follows, we will summarize and discuss our findings in light of the existing literature.

### 5.1 The use of enactment for mocking

Concerning the ways in which enactment can be used for mocking, a first finding relates to the creation of contrast. Our analysis shows that participants enact a target for the purpose of mockery (of either themselves or others) in an exaggerated or otherwise highly stylized way resulting in a contrast. This contrast could concern an incongruence between expectation and reality, or other norms to be adhered to, as is the case in Examples 1 and 5. In these examples, the interactants denounce specific characteristics of the target of the mockery by foregrounding and reshaping them in the way they represent these characters during enactments. However, as we saw in Examples 2 and 3, enactments can also be employed in a different way in mocking sequences. In these cases, the creation of the contrast does not lie in the design of the enactments themselves, in light of the expected or recognizable features of the target. Rather, these enactments serve to invoke a scenario that is mocked because of its absurdity.

A second finding concerns the use of viewpoint and character roles within the enactments. In prototypical cases of parodies to mock a target, the target is the character of the mockery, as in Examples 1, 2, 4, and 5. However, throughout the different data sets, we observed that signers/speakers also enact other characters that are not the target of the mockery, thus representing different perspectives, including their own viewpoint in response to the mocking enactment, or the viewpoint of other characters that are part of the depicted scenario (Examples 3 and 5). In other words, mocking enactments may not just consist of an enactment of the target but also of other characters who react to the target and stance object. This means that the target of the mockery, the stance object, and the enacted character do not necessarily overlap. In fact, a sequence with multiple enactments including viewpoint shifts, showing stances from different characters, can be employed to highlight contrasting stances.

### 5.2 The stance triangle in relation to stance-stacking

Through mocking enactments, stance objects are introduced on a non-serious level, outside of the shared base layer of the interaction, which results in a layering of evaluations and thereby a stacking of stances: In Examples 1 and 2, this was accomplished by introducing elaborate imagery embedded into a larger evaluation sequence, in Example 3 by invoking a (supposedly) absurd hypothetical scenario, and in Examples 4 and 5 by reporting on other characters’ actions and stances.

Along with the layering of stance objects as well as evaluations, positioning can take different forms in mocking enactments. The question “who enacts whom in order to mock whom” is central to positioning through mocking enactment and links to what we summarized above about the use of different viewpoints in enactments for mocking. Variation occurs depending on whether the current signer/speaker positions themselves as the target of the mocking (Example 2), another co-present participant (Examples 1, 3, and 5), or an (imagined) external individual (Examples 4 and 5). Depending on the viewpoint and referent depicted in the enactment, this positioning can occur directly or by extension through the abovementioned layering of object and evaluation.

The layering of evaluations as well as positioning also has an effect on the negotiation of alignment. As discussed in the existing literature, the negotiation of alignment unfolds temporally across turns in interaction and should be regarded as a continuum rather than a dichotomy (Iwasaki, 2022). In the case of mocking enactments, an opportunity for (dis)alignment is opened up at one specific moment in time, where several layers are presented to the addressee to act upon. As mentioned in Sections 2.2 and 4.3, this links Clark’s (1996, p. 360) distinction between imagination and appreciation. While the enacted scene can very well be imagined and aligned with on an epistemic or evidential level by the addressee of the mocking enactment, alignment with the mocking stance is not an obligatory consequence, which connects to the findings of Stivers (2008) on the difference between aligning and affiliating. In the case of layered stance acts, thus, participants can (dis)align, as well as partially (dis)align with the stance expressed (Holt, 2016).

Through the expression of stances on multiple interactional layers, interactants create stacked stances, with implications on every component of the Stance Triangle. Let us, therefore, zoom out to discuss what the model proposed by Du Bois has to offer for multimodal analyses of complex stance acts and longer stretches of interaction. In the existing literature on stance-taking, Du Bois’ triangle has been much discussed and critically reflected on (Debras, 2015; Thompson, 2016; Iwasaki, 2022). Several authors have already drawn attention to the limitations of the model and suggested re-interpretations, such as Debras (2015) in the study on constructed dialog. Because of the additional subject that is introduced through enactment which lies outside of the interaction, she suggests to re-interpret the triangle as a tetrad. While this presents a relevant extension to the existing model for the phenomenon studied in said article, we suspect that for any given phenomenon and the changing complexity in terms of layering and inclusion of more or fewer subjects or objects, no geometrical form would suffice to account for the whole constellation and temporal unfolding.

Iwasaki (2022) highlighted the temporal transcendence of stance-taking in interaction and the constant evolution of all components of the Stance Triangle with every new stance act that is expressed. In this contribution, we add onto that by showing how during one stance act, several layers can be at play on the different components of the triangle, which can then either in part or as a whole be picked up in subsequent discourse. In that sense, we understand the Stance Triangle as a snapshot of a given stance act, or usage event, at one level of the interaction. Conceiving the Stance Triangle as a snapshot, a brief manifestation of complex processes that will already have evolved an instant later allows, as we have shown in our analysis, to tease apart participant roles, the parallel processes of evaluation, positioning, and alignment, and therefore, to distinguish layering on any of the vertices or sides of the triangle. Hence, in a mocking enactment, several triangles overlap, for the serious and the non-serious layer and possibly another for a reported stance within the enactment, resulting in stacked stances. We argue that these layers can only be teased apart fully with a multimodal approach, taking into account the full potential of interactional mechanisms at language users’ disposal.

Rather than expanding the Stance Triangle, we, therefore, suggest taking it for what it is: a powerful model that breaks down complex cognitive, social, and interactive processes into a set of three components and processes, but which is inevitably limited and can never paint the full picture of stance-taking in complexly layered ways.

### 5.3 Multimodal design of mocking enactments

The third research aim concerned the multimodal design of mocking enactments. In this regard, we found that the enactments themselves were constructed multimodally, with a specific role for manual gestures in depicting the scene (e.g., clenched fists that open up in Example 1, or pointing to high locations to index flags in Example 5), in concert with many other resources (e.g., head tilts or shakes, eyebrow raises, and smiling) that have previously been described as having stance-related functions (see Section 2.1).

The multimodal design of individual enactments emerges locally in interaction. Therefore, it is difficult to identify specific patterns of multimodal resources that contribute to the layering of stances. Moreover, resources may serve multiple functions at the same time, which complicates assigning their use to the functions of, for example, either mocking or enacting (Cantarutti, 2021, p30–31). In Example 3, for instance, one of the interactants tilts her head during enactment, which can be an expression of her own stance on the enactment, a means to draw visual attention to the enactment, as well as a part of the enacted scene (see Figure 7 - Image 10). The use of multiple resources, however, constitutes a composite meaning [i.e., a multimodal “gestalt” (Mondada, 2014)], which results in a locally constructed stacked stance as a whole.

Furthermore, we found that mocking enactments are embedded in highly evaluative contexts that are constructed multimodally (e.g., gasping in Example 5, laughing in Examples 2 and 3, or covering the face with the hands in Example 3). As a result, a mocking enactment can often only be interpreted as a layered stance in relation to the preceding and succeeding interaction. We observed that in many cases, a negative evaluation has already been expressed or at least projected prior to the mocking enactment. In other words, one layer of the stacked stance may already be established before interactants add a second layer. This stacking can happen within the enactment but does not always have clear borders. In the context of the music instruction, for instance, the grammatical slot opener for the enactment space (“da’s zo,” *that’s like*, Example 1) usually projects a negative evaluation which in that case is expressed as an enactment of exaggerated imagery.

Although the prototypical case of a mocking enactment consists of an exaggerated or otherwise “distorted” multimodal depiction of the target (as in Examples 1 and 5, cf. D’Errico and Poggi, 2016), we found that this is not always the case (as in Examples 2 and 4). Both these enactments were not performed using large gestures, a higher number, or more animated use of semiotic resources, and were not “distorted” in any other way. Instead, in these cases, the enactments merely seemed to allude to the hypothetical scenario that is mocked. We believe that this warrants further investigation. In what cases do participants choose to produce an enactment with more ‘multimodal intensity’, and in what cases is the enactment more minimal? A first step in this direction would be to quantify what precisely constitutes ‘multimodal intensity’ as there is currently no established operationalization of this phenomenon. Regarding the question of what influences the multimodal intensity of the mocking enactments, one possible answer would be the following. The easier it is to tease apart the non-serious layers (e.g., by the use of more or specific multimodal marking), the less aggressive a mocking enactment is for a recipient, and conversely, the more ambiguous the mockery is, the more negative response it may receive (Keltner et al., 2001; Yu, 2013). Research with an experimental setup could shed light on this topic.

Additionally, while enactment serves as a tool for local stance-taking in interaction, it simultaneously contributes to the construction of larger identity categories, including social and cultural identities, influencing individuals’ perceptions of self and others both within and beyond immediate interactions. In these cases, an enacted individual can serve as a placeholder for a social group when interactants draw on stereotypes to mock these targets or their behavior. As in Example 5, a mocking enactment of an individual who goes out to party after a soccer game positions a whole social group as a target. The signer who produces this mocking enactment thereby excludes herself from this group and associated identities. While it is clear that mocking enactments contribute to the identity construction of self and others (Fischer and Simon, 2016; Gilbert, 2018; Van De Mieroop, 2020), both locally and beyond the borders of an interaction, the relation between these two dimensions remains largely unexplored. Cross-cultural and cross-linguistic research may uncover how mocking enactments are used to construct identities and (distancing) stances on a larger sociocultural scale.

## 6 Conclusion

The current contribution shows that mocking enactments offer fruitful grounds for the investigation of layered and stacked stances. We explored the use of enactment in mockery, finding that participants enact various characters, not only their targets but also incorporating other viewpoints. The shifts in perspective and highlighting of stacked and often contrasting stances show that the enacted character, the target of the mockery, and the stance object may not necessarily overlap. While acknowledging the limitations of the Stance Triangle (Debras, 2015; Iwasaki, 2022), we view it as a useful framework to approach even intricately layered stance acts. Based on this model, we have shown that mocking enactments go together with layering on all components of the triangle, representing the base layer of interaction as well as potential non-serious and enacted layers. Furthermore, mocking enactments are embedded in highly evaluative contexts, which are indexed by a plethora of resources (bodily visual and/or vocal-aural, generic, or setting-specific). Finally, we found that although enactments *can* be staged in an exaggerated or highly stylized way, this is not necessarily always the case, an observation that warrants further (experimental) research.

## Data availability statement

The datasets presented in this article are not readily available because the data analyzed in this study are subject to the following licenses/restrictions: The Corpus Flemish Sign Language is publicly accessible via www.corpusvgt.ugent.be. The other raw video data used in this study cannot be made available for privacy reasons. Anonymized fragments referred to in the analysis are available in the Supplementary material. Requests to access the datasets should be directed to FA, fien.andries@kuleuven.be.

## Ethics statement

The studies involving humans were approved by Social and Societal Ethics Committee - KU Leuven. The studies were conducted in accordance with the local legislation and institutional requirements. The participants provided their written informed consent to participate in this study. Written informed consent was obtained from the individual(s) for the publication of any potentially identifiable images or data included in this article.

## Author contributions

CdV: Writing – original draft, Writing – review & editing. FA: Writing – original draft, Writing – review & editing. KM: Writing – original draft, Writing – review & editing.

## Footnotes

^1^Within this study, we view stance, enactment, and mocking as multimodal and polysemiotic phenomena as they may involve different perceptual modalities and semiotic systems. For an elaborate discussion of the use of the terms polysemiotic and multimodal, we refer the reader to Zlatev (2019).

^2^For the brainstorms, participants received the instruction to talk about their ideal student house and bar. In practice, this resulted in spontaneous discussions of houses that participants lived in and bars that they went to.

^3^For the spoken language examples, GAT2 conventions were used for the transcriptions of speech (Selting et al., 2009), and Mondada’s (2018) conventions for transcribing multimodal conduct.
